# Longitudinal metagenomic profiling of bovine milk to assess the impact of intramammary treatment using a third-generation cephalosporin

**DOI:** 10.1038/srep37565

**Published:** 2016-11-22

**Authors:** Erika K. Ganda, Rafael S. Bisinotto, Svetlana F. Lima, Kristina Kronauer, Dean H. Decter, Georgios Oikonomou, Ynte H. Schukken, Rodrigo C. Bicalho

**Affiliations:** 1 Department of Population Medicine and Diagnostic Sciences, College of Veterinary Medicine, Cornell University, Ithaca, NY, USA; 2 Epidemiology and Population Health, Institute of Infection and Global Health, University of Liverpool, Liverpool, UK

## Abstract

Antimicrobial usage in food animals has a direct impact on human health, and approximately 80% of the antibiotics prescribed in the dairy industry are used to treat bovine mastitis. Here we provide a longitudinal description of the changes in the microbiome of milk that are associated with mastitis and antimicrobial therapy. Next-generation sequencing, 16 S rRNA gene quantitative real-time PCR, and aerobic culturing were applied to assess the effect of disease and antibiotic therapy on the milk microbiome. Cows diagnosed with clinical mastitis associated with Gram-negative pathogens or negative aerobic culture were randomly allocated into 5 days of Ceftiofur intramammary treatment or remained as untreated controls. Serial milk samples were collected from the affected quarter and the ipsilateral healthy quarter of the same animal. Milk from the mastitic quarter had a higher bacterial load and reduced microbial diversity compared to healthy milk. Resolution of the disease was accompanied by increases in diversity indexes and a decrease in pathogen relative abundance. *Escherichia coli*-associated mastitic milk samples had a remarkably distinct bacterial profile, dominated by Enterobacteriaceae, when compared to healthy milk. However, no differences were observed in culture-negative mastitis samples when compared to healthy milk. Antimicrobial treatment had no significant effect on clinical cure, bacteriological cure, pathogen clearance rate or bacterial load.

Production of animal protein to support the world’s growing human population is one of the main challenges facing humankind. Concerns related to food safety and development of antimicrobial resistance may lead to decreased availability of antibiotics for use in food animals and thereby limit our ability to control disease in agricultural animal species. Such a change in antibiotic usage in food animals could also alter perspectives on food safety and security as it relates to public health concerns regarding antibiotic use in food animals. Thus, in-depth understanding of disease mechanisms is critical to promote animal health and at the same time encourage judicious use of antibiotics in livestock. Mastitis is one of the most common diseases in dairy herds, and approximately 20% to 30% of dairy cows develop clinical mastitis at least once during lactation^1^,^2^. Not surprisingly, prophylaxis and treatment of mastitic cows are the major reasons for antibiotic usage in dairy farms^3^,^4^.

Maternal milk harbors a rich microbial community that is vital for establishment of the gut microbiome and immune tolerance in neonates^5^,^6^. The same microbial community in the mammary gland may provide an environment that aids the host in preventing mammary infections. For instance, commensal bacteria present in human milk inhibit proliferation of *Staphylococcus aureus*^7^, which is also a pathogen commonly associated with mastitis in dairy cows^8^. Considering that mastitis possibly reflects a dysbiosis within the mammary gland^9^,^10^,^11^, a detailed assessment of the milk microbiome during active stages of clinical disease, spontaneous recovery, treatment and post-treatment is essential to further elucidate this pathological condition.

The multifactorial etiology of mastitis presents a major challenge for disease prevention and treatment of affected animals. Implementation of programs for mastitis control has reduced the prevalence of important contagious pathogens, and approximately 40% of clinical cases of mastitis are associated with opportunistic Gram-negative bacteria such as *Escherichia coli, Klebsiella* spp., *Pseudomonas* spp., and *Pasteurella* spp.^8^,^12^,^13^. Although current guidelines do not recommend the use of intramammary antibiotics for cows diagnosed with Gram-negative mastitis^3^,^14^, improved bacteriological and clinical outcomes have been shown in mastitic cows treated with third-generation cephalosporins compared with other antimicrobials or untreated controls^15^,^16^. However, the impact of these broad-spectrum antibiotics on the milk microbiota (other than major pathogens) remains unknown. In fact, currently, no data concerning the effect of antibiotic therapy on the mammary microbiota are available in either humans or animals. Routine methods used to assess responses to intramammary treatments overlook numerous microorganisms, which is supported by the fact that 40% of milk samples collected from cows with clinical mastitis yield negative results by aerobic culture^13^. Later-generation cephalosporins have broad-spectrum antibacterial activity, and their use could unintentionally disrupt general mammary microbial populations and also increase the risk of antimicrobial resistance if not used in a judicious manner^17^. Understanding the dynamics of the mammary microbiota upon antibiotic therapy is essential not only for development of effective treatment strategies, but also to facilitate the process of restoring a healthy mammary microbiota.

State-of-the-art technologies have allowed the investigation of microbial communities in milk without some of the limitations imposed by culture methods^9^,^18^,^19^. Therefore, the specific objectives of the present study were: 1) to use high-throughput DNA sequencing to describe the microbiome of milk in dairy cows affected by clinical mastitis associated with Gram-negative bacteria or negative culture; 2) to determine the bacterial load based on PCR quantification of 16S rRNA gene copies, and compare microbial populations of affected and healthy quarters; and 3) to assess the effect of intramammary treatment with ceftiofur hydrochloride on the milk microbiome, bacterial load, and clinical cure in quarters affected with clinical mastitis.

## Methods

### Ethics Statement

The research protocol was reviewed and approved by the Cornell University Institutional Animal Care and Use Committee (protocol number 2013-0056). The methods were carried out in accordance with the approved guidelines.

### Animals, Enrollment Criteria, and Treatments

Milk samples were collected from lactating Holstein cows diagnosed with clinical mastitis between April and June, 2014. All cows were housed in a single herd located in upstate New York which milked approximately 2,800 cows thrice daily during the experimental period. Clinical mastitis was defined as the presence of at least visually abnormal milk (i.e. presence of flakes, clots, or serous milk) during forestripping performed at the milking parlor. Once mastitis was diagnosed, the initial milk sample for mastitis pathogen identification was collected by trained farm personnel according to National Mastitis Council guidelines. These samples were defined as day 0 samples. Teats were cleaned and disinfected using 70% ethanol (v/v), the initial three streams were discarded, and approximately 5 mL of milk was collected into a sterile plastic tube without preservative (Corning Life Sciences, Tewksbury, MA). Milk samples were cultured using an on-farm chromogenic culture system for fast identification of causal agents (Accumast^®^, FERA Animal Health LCC, Ithaca, NY) according to the manufacturer’s recommendations, and then submitted for analysis at the Quality Milk Production Services laboratory (**QMPS**; Cornell University, Ithaca, NY) to ensure the accuracy of on-farm culture. Disagreement between methods was observed in only two samples, which were excluded from further analyses after the results from QMPS were received.

Cows diagnosed with clinical mastitis associated with Gram-negative bacteria or negative on-farm culture and that had not been treated with intramammary antimicrobials in the 14 days preceding diagnosis were deemed eligible for enrollment. On study day 1, eligible cows were randomly allocated into one of two treatments based on a list of numbers generated using the RAND function of Excel (Microsoft, Redmond, WA), blocked by aerobic culture results. Clinical score was assessed on days 1, 8, 10 and 14 according to Wenz *et al*.^20^,^21^. Milk appearance, mammary gland appearance and systemic signs of disease (i.e. rectal temperature ≥39.5 °C, dehydration and depression) were evaluated for classification of clinical score. A clinical score of ‘mild’ was assigned if only abnormal milk was observed. A ‘moderate’ score was assigned when abnormal milk and inflammation of the mammary gland were present. A ‘severe’ score was assigned if abnormal milk, local inflammation and one or more of the systemic signs of the disease were observed.

Cows assigned to the treatment group received daily intramammary infusions containing 125 mg of ceftiofur equivalents (as ceftiofur hydrochloride; Spectramast LC^®^, Zoetis, Florham Park, NJ) only on the mastitic quarter for five consecutive days, whereas those assigned to the control group did not receive intramammary therapy.

### Sample and Data Collection

Serial milk samples were collected by a trained veterinarian member of the research team from each cow on study days 1, 2, 3, 4, 5, 8, 10, and 14, from both the mastitic quarter and the ipsilateral healthy quarter of the same cow. For cows in the treated group, sampling on days 1 through 5 was performed after milk out of the quarter in untreated cows, whereas treated cows were sampled immediately before intramammary treatments were applied. Teats were disinfected as described above and 10 mL of milk was harvested from each quarter into a sterile plastic tube without preservative (Corning Life Sciences, Tewksbury, MA). Samples were immediately refrigerated at 4 °C, transported to the laboratory on ice, and frozen at −20 °C until assayed. Milk samples collected from mastitic quarters on days 10 and 14 were submitted to the QMPS laboratory for bacterial identification using standard aerobic culture.

Clinical cure was defined as cows without any clinical signs on both day 10 and day 14. Bacteriological cure was defined as both the samples taken on day 10 and day 14 being negative for the organism present on day 0. In all other cases the quarter was considered to be a non-cure or treatment failure. Bacteriological cure can only be evaluated in quarters that were culture positive on day 0.

### DNA Isolation and Purification

Milk samples were thawed, homogenized by inverting the tubes, and a 6-mL aliquot was taken for DNA isolation and purification. Milk samples were centrifuged at 4 °C and 16,100 × *g* for 3 minutes and the supernatant was discarded. Genomic DNA was isolated from the remaining pellet using a commercially available kit (PowerFood DNA Isolation Kit, MO BIO Laboratories Inc., Carlsbad, CA) as described previously^22^. Concentration and purity of isolated DNA were evaluated based on optical density at 230, 260 and 280 nm wavelengths (NanoDrop ND-1000, NanoDrop Technologies, Wilmington, DE).

### Amplification of the V4 Hypervariable Region of the Bacterial 16S rRNA Gene, Library Preparation, and 16S rRNA Gene Sequencing

The V4 hypervariable region of the bacterial 16S rRNA gene was amplified from genomic DNA by PCR utilizing the primers 515F and 806R optimized for the Illumina MiSeq platform (Illumina Inc., San Diego, CA)^23^ as described previously^24^.

Equimolar libraries were sequenced in six runs using the MiSeq reagent kit V2 for 300 cycles on the MiSeq platform (Illumina). Each run included 279 samples and a sequencing control that consisted of the purified barcoded PCR product of DNA extracted from *Staphylococcus aureus* (ATCC 25923). Gene sequences were processed using the 16S Metagenomics workflow in the MiSeq Reporter analysis software version 2.5 based on quality scores generated by real-time analysis during the sequencing run. Quality-filtered indexed reads were demultiplexed for generation of individual FASTQ files and aligned using the banded Smith-Waterman method of the Illumina-curated version of the Greengenes database for taxonomic classification of milk microbes. Resulting FASTQ files were uploaded into the open-source pipeline Quantitative Insights into Microbial Ecology (**QIIME**) version 1.9.1^25^. Sequences were filtered based on quality as described previously^26^ and assigned to operational taxonomic units (**OTUs**) with 97% identity using UCLUST^27^. The OTU database was rarefied using the command single_rarefaction.py from QIIME and the number of OTUs, as well as Chao1 and Shannon indexes, was calculated for each sample at a rarefication level of 5,000 reads per sample.

### Quantification of 16S rRNA Copies by qPCR

The number of 16S rRNA copies was used as a proxy to determine bacterial load in milk samples collected on days 1, 3, 8 and 14. 16S rRNA gene copies were quantified by qPCR as described previously^28^. Reactions were performed using Unibac primers (forward: 5′-TGG AGC ATG TGG TTT AAT TCG A-3′; reverse: 5′-TGC GGG ACT TAA CCC AAC A-3′; 50 pmol/reaction), 1X iQ^TM^ SYBR^®^ Green Mastermix (Bio-Rad Laboratories, Hercules, CA), and 1.5 μL of sample DNA. A standard curve was built using plasmid DNA quantified by spectrophotometry. All samples were assayed in duplicate using an iQ^TM^5 Real-time PCR system (Bio-Rad Laboratories, Hercules, CA) set to perform denaturation at 95 °C for 3 minutes, 40 cycles of amplification (95 °C for 10 seconds and 55 °C for 30 seconds), one cycle at 95 °C for 60 seconds, one cycle at 55 °C for 60 seconds, and a melting curve determination.

### Statistical Analyses

Descriptive analyses on sequencing results were performed using the UNIVARIATE procedure of SAS version 9.3 (SAS Institute Inc., Cary, NC). Differences in the relative abundance of bacteria between quarters with clinical mastitis and healthy counterparts were evaluated at the phylum and family levels using JMP Pro 11 (SAS Institute Inc., Cary, NC). Cows were categorized according to the main pathogen identified on samples taken on study day 0 through standard culture methods into four groups, namely *E. coli, Klebsiella* spp., *Pseudomonas* spp., and negative culture. Within each group, the effect of clinical mastitis on the relative abundance of each of the ten most prevalent phyla was evaluated by ANOVA. The prevalences of all remaining phyla were combined into a single cluster. The fixed effect of disease (healthy vs. mastitic quarters) was included in the statistical models as an independent variable. Response screening was performed to assess the effect of clinical mastitis on the relative abundance of the 100 most prevalent families in each pathogen group. *P*-values were adjusted for false discovery rate (**FDR**^29^) and presented as FDR LogWorth (i.e. −log_10_*P*). The mean relative abundance for each family observed across all healthy quarters was used as a reference for calculation of fold-changes.

Microbiome changes occurring over time and in response to intramammary antibiotic therapy were described for the 25 most prevalent families in each pathogen group using the tabulate function of JMP Pro 11. Relative abundances of all remaining families were combined into a single cluster. The magnitude of change was scaled uniformly within health status (healthy vs. mastitic quarters). The relative abundances of major pathogens associated with clinical mastitis were evaluated within pathogen groups by ANOVA for repeated measures using the GLIMMIX procedure of SAS. Outcomes were the relative abundance of each pathogen and the explanatory variables were treatment, time, health status (healthy vs mastitic quarter) and their two- and three-way interactions. Cow was considered a random effect in all statistical models. Time changes in the number of OTUs, Chao1 index, and Shannon index were analyzed by ANOVA for repeated measures using the GLIMMIX procedure of SAS. Within each pathogen group, two statistical models were built to evaluate the effects of mastitis (i.e. fixed effects of mastitis, time, and interaction between mastitis and time) and treatment (i.e. fixed effects of treatment, mastitis, time, and all two- and three-way interactions).

The effect of cure on the relative abundance of Enterobacteriaceae family members and the Shannon diversity index was evaluated between cured and non-cured cows with clinical mastitis associated with *E. coli* by ANOVA for repeated measures using the Fit Model function on JMP Pro 11. Tests for normality of residuals and homogeneity of variances were conducted for each dependent variable, and data that did not fulfill ANOVA assumptions were transformed accordingly (i.e. 16S rRNA gene copy numbers). The covariance structure with the smallest Schwarz’s Bayesian information criterion value was selected for each analysis. Differences with *P* ≤ 0.05 were considered significant and those with 0.05 < *P* ≤ 0.10 were considered tendencies. Results are presented as average and standard deviation (i.e. descriptive analyses of sequencing results) or least square means followed by the respective standard error of the mean.

Multivariate analysis of microbiome data was carried out in R (R Core Team, Vienna, Austria)^30^ and QIIME. Beta diversity was analyzed through analysis of similarities (**ANOSIM**) using non-rarefied data normalized employing the packages metagenomeSeq^31^ and vegan^32^ in R. Principal coordinate analysis (**PCoA**) was performed using weighted Unifrac distances calculated in QIIME and visualized through EMPeror^33^.

## Results

### Clinical and Bacteriological Cure

Intramammary treatment with ceftiofur hydrochloride did not significantly improve clinical and bacteriological cures of clinical mastitis compared with untreated controls (Table 1). Of the 40 cows enrolled in the control group, 75% (n = 30) experienced clinical cure, whereas of the 40 cows that received intramammary antibiotic therapy, 77.5% (n = 31) experienced clinical cure (*P-*value = 0.79). Clinical cures for cows affected with Gram-negative intramammary infections also did not differ between the treated (75% cure rate) and control cows (73.9% cure rate) (*P-*value = 0.93). Bacteriological cure followed the same pattern as for clinical cure, with 82.6% of the milk samples collected from non-treated mastitic quarters being negative on days 10 and 14 for the organism present on day 0, whereas in the treated group, 79.2% of the samples were considered to be bacteriological cures (*P*-value = 1.00). Bacteriological cure was not altered by treatment when the data were stratified and analyzed by each pathogen group (Table 1).

### Real-time PCR Results

**Figure 1:**
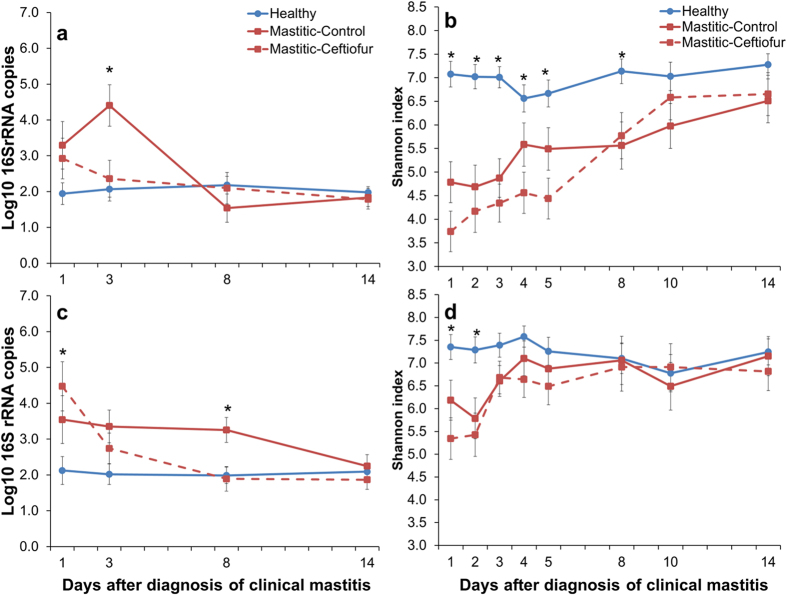
Effect of clinical mastitis and intramammary treatment with ceftiofur hydrochloride (days 1–5) on the number of 16S rRNA gene copies in cows with clinical mastitis associated with *Escherichia coli* (**a**) or negative culture (**c**), and microbial diversity in cows with clinical mastitis associated with *Escherichia coli* (**b**) or negative culture (**d**). Bars represent standard error of the mean. Asterisks represent significant differences at α = 0.05 between groups within the same study day. (**a**) Mastitic-Control had a significantly greater bacterial load than Mastitic-Ceftiofur and healthy quarters on day 3. (**c**) On day 1, both mastitic quarters had a significantly greater bacterial load when compared to healthy quarters. On day 8, Mastitic-Control had a significantly greater bacterial load than Mastitic-Ceftiofur and healthy quarters.

Cows diagnosed with clinical mastitis caused by *E. coli* had a significantly (*P* = 0.008) lower number of 16S rRNA gene copies in healthy quarters compared to mastitic ones on day 3 post diagnosis; however, no difference was observed in the bacterial load as measured in 16S rRNA gene copies between healthy and mastitic quarters at days 8 and 14. Intramammary treatment with Ceftiofur caused a significant decrease in the bacterial load of mastitic quarters on day 3 (*P* = 0.01) compared to non-treated mastitic quarters. Nonetheless, a treatment effect was no longer observed at study day 8 (Fig. 1a).

In animals diagnosed with clinical mastitis yielding no bacterial growth upon aerobic culture, the number of 16S rRNA gene copies was higher in mastitic quarters compared to healthy ones. No treatment effect was observed on the bacterial load in this group of animals. Mastitic and healthy quarters exhibited the same bacterial load by study day 14 (Fig. 1c).

### Sequencing Results

Quality-filtered reads were demultiplexed and a total of 67,413,334 sequences was used for downstream analyses (mean = 47,241.3 ± SD = 32,625.0 reads/sample). The median length for all reads was 301 bp.

### Microbiome Changes Associated With Clinical Mastitis

**Figure 2:**
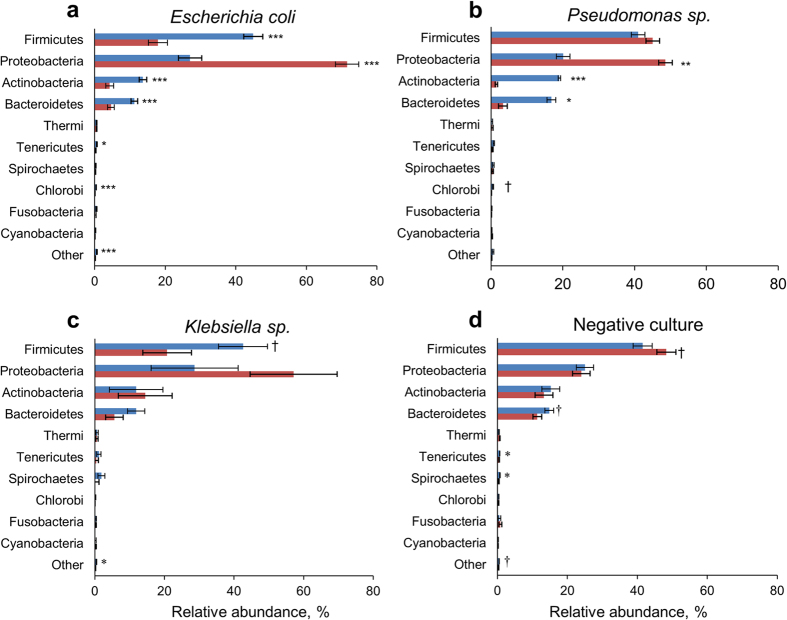
Relative abundance of phyla in quarters diagnosed with clinical mastitis (red bars) and healthy quarters (blue bars) according to identification of milk pathogens by laboratory culture. ^***^P ≤ 0.001, ^**^P ≤ 0.01, ^*^P ≤ 0.05, ^†^*P* ≤ 0.10. Bars represent standard error of the mean.

**Figure 3:**
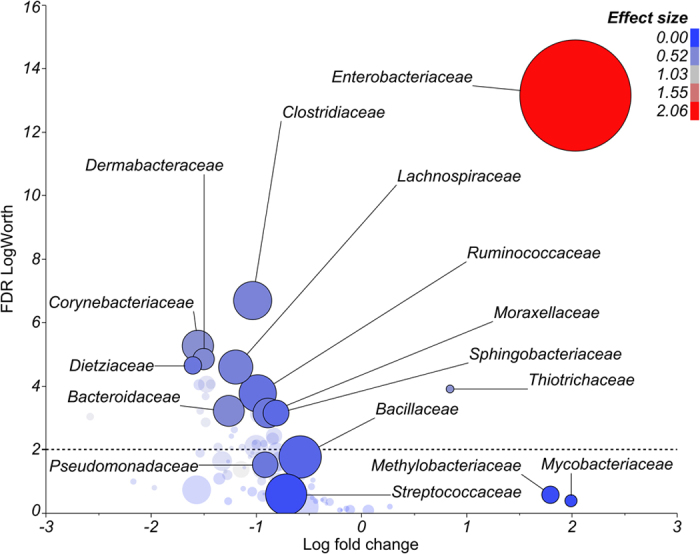
Comparison of the microbiome from quarters with clinical mastitis associated with *Escherichia coli* and healthy quarters (i.e. reference for calculation of fold change). Size of the circle is proportional to the overall prevalence of each family. Color of the circle is associated with effect size. The graph plots log fold change in 16S rRNA gene abundance in mastitic relative to healthy control quarters versus false discovery rate (FDR) logWorth (i.e. −log10P). P-values are adjusted for FDR. The dashed line represents the adjusted *P*-value = 0.01.

The mean relative abundance of bacteria from the phylum Proteobacteria was greater (*P* < 0.01) in the milk from mastitic quarters infected by *E. coli* and *Pseudomonas* spp. compared with that of healthy quarters (Fig. 2a,b). This was driven mostly by greater abundances of Enterobacteriaceae (*P* < 0.001; Fig. 3) and Pseudomonadaceae (*P* = 0.03; Fig. S1). On the other hand, the average abundance of Firmicutes, Actinobacteria, Bacteroidetes, Tenericutes, Chlorobi, and the combination of all remaining phyla was greater (*P* < 0.05) in healthy compared with mastitic quarters infected by *E. coli* (Fig. 2a). A similar pattern was observed in cows with clinical mastitis associated with *Pseudomonas* spp. (n = 2 quarters), in which the abundance of Actinobacteria and Bacteroidetes was greater (*P* < 0.05) and that of Chlorobi tended to be greater (*P* = 0.08) in healthy compared with mastitic quarters (Fig. 2b). The diversity of milk microbial populations was reduced (*P* < 0.0001) in *E. coli* mastitis compared with healthy quarters (Fig. 1b). The Shannon index was also influenced (*P* < 0.0001) by the interaction between mastitis and time, as values increased from day 1 through 14 in mastitic quarters, whereas no change was observed in healthy counterparts (Fig. 1b). Likewise, the richness of microbial communities was reduced in *E. coli* mastitis compared with healthy quarters (Fig. S2a).

The relative abundances of Firmicutes (*P* = 0.06) and the remaining phyla (*P* = 0.02) were greater in healthy quarters compared with those infected by *Klebsiella* spp. (Fig. 2c). Nevertheless, mastitis did not affect the relative abundances of other phyla or individual families (Fig. S3). Clinical mastitis associated with *Klebsiella* spp. had reduced (*P* = 0.05) Shannon values shortly after diagnosis but had no effect on the Chao1 index (Fig. S4a,b).

**Figure 4:**
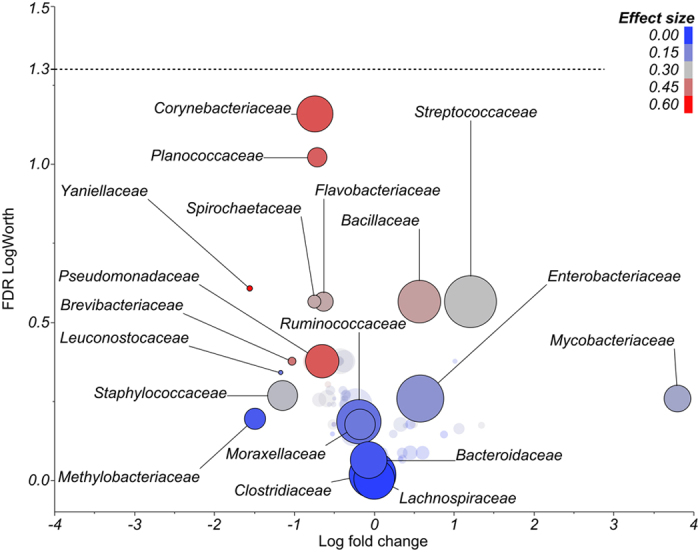
Comparison of the microbiome from quarters with clinical mastitis associated with negative culture and healthy quarters (i.e. reference for calculation of fold change) on day 0. Size of the circle is proportional to the overall prevalence of each family. Color of the circle is associated with effect size. The graph plots log fold change in 16S rRNA gene abundance in mastitic relative to healthy control quarters versus false discovery rate (FDR) logWorth (i.e. −log10P). P-values are adjusted for FDR. The dashed line represents the adjusted *P*-value = 0.05.

Shifts in the milk microbiome were less pronounced in cases of clinical mastitis associated with a negative aerobic culture (Fig. 2d). The relative abundance of Firmicutes was higher (*P* = 0.08), whereas those of Bacterioidetes (*P* = 0.06), Tenericutes (*P* = 0.05), Spirochaetes (*P* = 0.01), and the combined remaining phyla (*P* = 0.08) were lower in mastitic quarters. Fluctuations in bacterial communities were not associated with specific families (Fig. 4). Nevertheless, diversity was reduced (*P* < 0.01) in mastitic compared with healthy quarters (Fig. 1d). The same trend was observed in the Chao1 richness index on the first two days after diagnosis of clinical mastitis (Fig. S2b).

### Effect of Intramammary Antibiotic Therapy on the Milk Microbiome

**Figure 5:**
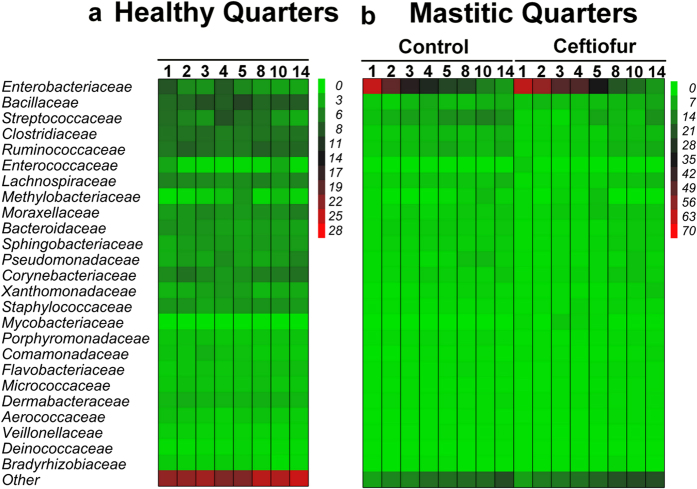
Effect of intramammary treatment with ceftiofur hydrochloride on relative abundance of the 25 most prevalent families in milk from quarters with clinical mastitis associated with *Escherichia coli*. Numbers indicate day after diagnosis of clinical mastitis.

**Figure 6:**
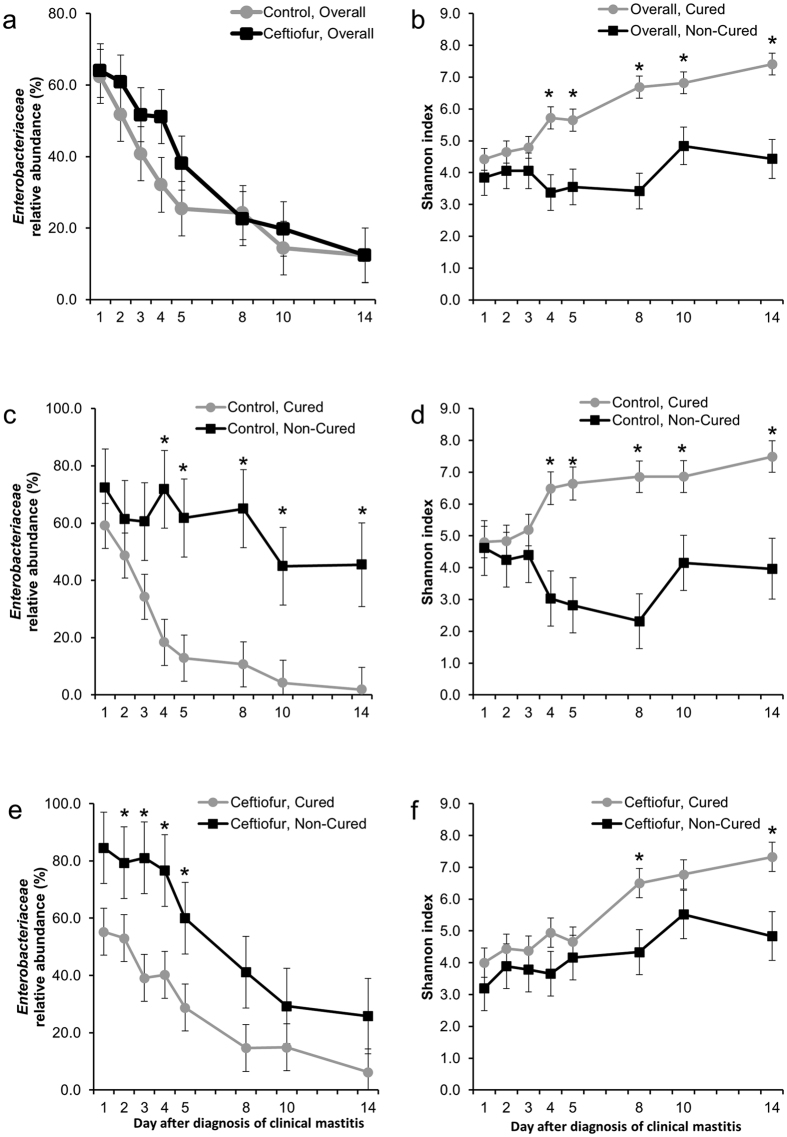
Effect of intramammary treatment with ceftiofur hydrochloride (days 1–5) or cure on the relative abundance of Enterobacteriaceae and Shannon diversity index in cows with clinical mastitis associated with *Escherichia coli*. (**a**) Effect of intramammary treatment with ceftiofur hydrochloride (days 1–5) on the relative abundance of Enterobacteriaceae in cows with clinical mastitis associated with *E. coli*. Effect of eventual clinical cure on the relative abundance of Enterobacteriaceae in cows with clinical mastitis associated with *E. coli* on control cows (**c**) and treated cows (**e**). Effect of cure on the Shannon index in cows with clinical mastitis associated with *E. coli* (**b**), on control cows (**d**) and treated cows (**f**). Asterisks represent significant differences at α = 0.05 between groups within the same study day.

In cows diagnosed with clinical mastitis caused by *E. coli*, microbiome dynamics in healthy quarters did not change over time (Fig. 5a). On the other hand, the relative abundance of Enterobacteriaceae decreased from study day 1 to 14 (62.6% vs. 9.7%), whereas the relative abundances of other families increased in mastitic quarters (Fig. 5b). Changes in milk bacterial populations were not affected by intramammary therapy with ceftiofur hydrochloride (Fig. 5b). Treatment and the interaction between treatment and time did not affect the relative abundance of Enterobacteriaceae (Fig. 6a), or the Shannon (Fig. 1c) and Chao1 (Fig. S2a) indexes in mastitic quarters infected by *E. coli*.

Similar patterns were observed in cows with clinical mastitis caused by *Klebsiella* spp., as intramammary therapy did not impact the milk microbiome or the relative abundance of Enterobacteriaceae (Figs S5 and S6). The only two cows diagnosed with clinical mastitis associated with *Pseudomonas* spp. presented an elevated abundance of Pseudomonadaceae on day 1 (44.3%), which was reduced until day 8 (3.3%) and then returned to initial values on day 14 (46.1%). Because both cows were assigned to receive intramammary infusion with ceftiofur hydrochloride, the effect of treatment on abundance of *Pseudomonas* spp. could not be assessed.

**Figure 7:**
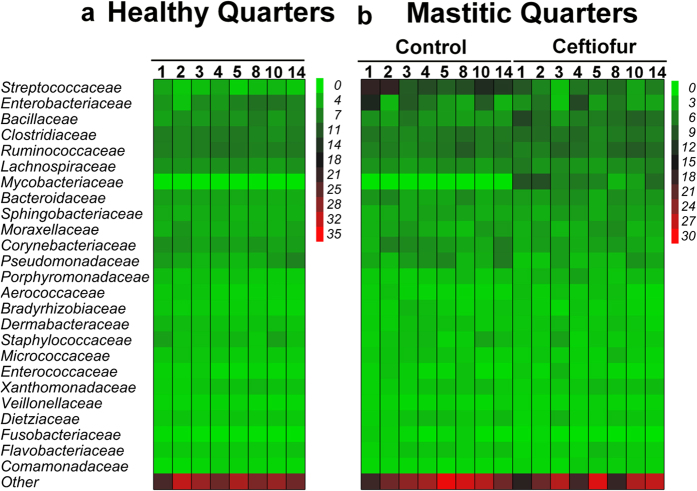
Effect of intramammary treatment with ceftiofur hydrochloride on relative abundance of the 25 most prevalent families in milk from quarters with clinical mastitis associated with negative culture. Numbers indicate day after diagnosis of clinical mastitis.

Changes in the milk microbiome over time were not observed in cows affected by clinical mastitis associated with negative aerobic culture (Fig. 7a). Moreover, intramammary treatment with ceftiofur hydrochloride in these quarters did not affect the milk microbiome (Fig. 7b) or the measures of microbial diversity and richness (Fig. 1d), (Fig. S2b).

### Microbiome Changes Associated With Clinical Mastitis Cure on the Mastitic Quarters of Cows With Mastitis Caused by *Escherichia coli*

In cows diagnosed with clinical mastitis caused by *E. coli*, microbiome dynamics in mastitic quarters exhibited remarkable changes over time. Quarters that experienced clinical cure by the end of the study period had significantly lower abundances of Enterobacteriaceae family members in both control (Fig. 6c) and treated animals (Fig. 6e). Nevertheless, microbial diversity at diagnosis of clinical mastitis did not differ between quarters that eventually became cured or not (Fig. 6b). However, microbial diversity of quarters that eventually were cured showed increasing microbial diversities in both the control (Fig. 6d) and treated groups (Fig. 6f) relative to quarters that did not show bacteriological cure during the study period. Similar patterns were observed for bacteriological cure (Fig. S7).

### Multivariate Analysis of Microbiome Data from Healthy and Mastitic Quarters

**Figure 8:**
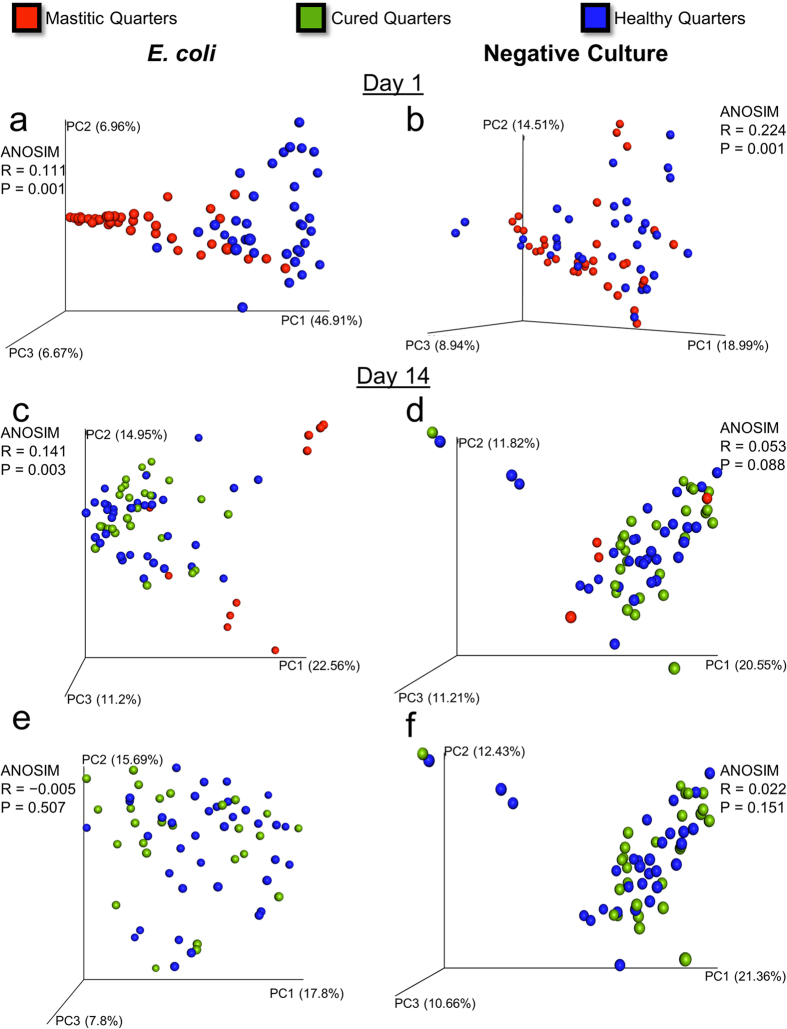
Principal coordinate analysis of weighted Unifrac distances and ANOSIM analysis comparing the microbiome data of samples from healthy and mastitic quarters on day 1 (**a** and **b**) and day 14 (**c, d, e** and **f**). Samples from quarters with clinical mastitis associated with *E coli* are depicted in sections (**a, c** and **e**). Samples from quarters with clinical mastitis associated with negative culture are shown in sections (**b**, **d** and **f** ).

Analysis of similarities revealed that mastitic quarters were significantly different from healthy quarters at the first day after diagnosis of clinical mastitis in cows with clinical mastitis associated with *E. coli* (Fig. 8a), and negative culture (Fig. 8b). A clear separation between mastitic and healthy quarters was observed in the principal coordinate analysis of weighted Unifrac distances in animals with clinical mastitis associated with *E. coli* (Fig. 8a); however the same could not be observed in animals with mastitis associated with negative culture (Fig. 8b). At the end of the study, namely day 14 after diagnosis of clinical mastitis, the microbiome of quarters that had been cured from clinical mastitis did not differ from the one of healthy quarters in either ANOSIM or Unifrac PCoA in cows previously identified with clinical mastitis associated with *E. coli* (Fig. 8e) nor in animals with clinical mastitis yielding negative aerobic culture (Fig. 8f). Interestingly, when the microbiome of quarters that remained with abnormal milk by the end of the study was included in the analysis, a significant difference could be observed in both ANOSIM and Unifrac PCoA on the microbiome of milk from cows identified with clinical mastitis associated with *E. coli* (Fig. 8c). No separation between mastitic, healthy, and cured quarters could be observed when the first three components of Unifrac PCoA were plotted in animals with mastitis associated with negative culture (Fig. 8d).

## Discussion

In an endeavor to better understand the effect of a third-generation cephalosporin (ceftiofur) in Gram-negative and culture-negative bovine mammary infections, we used high-throughput DNA sequencing to assess longitudinal changes in the microbiome of mastitic and healthy milk in a randomized clinical trial. Our data demonstrate that antimicrobial treatment did not significantly affect total bacterial load in the infected quarters by the end of the treatment period, nor was it able to increase the rate of pathogen clearance within the mammary gland. Moreover, this is the first study to document in depth the dynamics of the milk microbiota longitudinally using state-of-the art technology.

Treatment with ceftiofur did not affect clinical or bacteriological cure and did not have long-lasting effects on the milk microbiome. Our results are in disagreement with those of Schukken *et al*.^34^, who reported 38% bacteriological cure in non-treated cows and 73% bacteriological cure in treated animals. Their 5-day intramammary treatment regime with ceftiofur resulted in a significant increase in bacteriological cure, particularly in animals infected with *E. coli*, whereas our results demonstrate no difference between treated and untreated animals in this aspect. However, our results are in agreement with those of a landmark study conducted by Lago *et al*.^35^,^36^, which demonstrated that selective antimicrobial treatment of mastitic cows can lead to a considerable reduction in antimicrobial use without any significant differences in days to clinical cure, bacteriological cure risk, new intramammary infection risk or treatment failure. In that study, cows diagnosed with mastitis associated with *E. coli* either received two intramammary doses of cephapirin sodium 12 hours apart or did not receive antimicrobial treatment. The investigators were not able to detect any differences between treated and untreated animals in either clinical or bacteriological cures. It is important to acknowledge that the treatment applied in that study utilized a first-generation cephalosporin, which has a lower effectiveness against Gram-negative pathogens compared to ceftiofur^16^.

Despite the observed effect of intramammary infusion of ceftiofur in reducing the total bacterial load measured by qPCR of the V4 region of the 16sRNA gene in the affected quarter on day 3, we failed to detect any differences between treated and untreated quarters at days 8 and 14 post diagnosis. Furthermore, our data revealed that ceftiofur therapy had no effect on total bacterial load 3 days after cessation of treatment. We also assessed the longitudinal effect of antibiotic therapy on the relative abundance of the causal mastitis pathogens between treated and untreated cows; again, no differences were observed between the treatment and control groups. The observation that pathogen load was not affected by antimicrobial treatment is substantiated by a consistent decrease in the relative abundance of Enterobacteriaceae at 14 days post diagnosis in both treatment arms. Lastly, regardless of the treatment group, milk samples obtained on day 14 from all quarters deemed as mastitic on day 0 and that had normal milk on day 14 all presented a similar, more diverse bacterial profile, one remarkably comparable to that in healthy milk. Our data demonstrate that antimicrobial therapy does not improve cure rates for mastitis caused by *E. coli*, given the similar patterns of reduction in the percentage of pathogens over time in treated versus non-treated animals, which is in line with the results of Leininger *et al*.^37^ and the recommendations of Suojala *et al*.^14^.

We have demonstrated how the microbiome of mastitic quarters associated with Gram-negative pathogens dynamically changes over time. More interestingly, quarters that were not cured by the end of the study period had diverging abundances of Enterobacteriaceae and microbial diversities over time when compared to mastitic quarters that became healthy by the end of the study period. Reduced bacterial diversity has also been reported in other studies comparing samples derived from healthy and diseased mammary environments^9^,^11^,^38^. Although, most mastitis cases caused by *E. coli* are of an acute/peracute nature and have a high self-cure rate, chronic cases have been reported in the literature^39^,^40^. Further research is needed to understand the host and pathogen idiosyncrasies that are associated with the chronification of these *E. coli*-related mastitis cases.

Elucidating the milk microbiome has been a daunting task^41^, particularly in clinical mastitis with negative culture results^10^. Various reasons could explain why a negative result might be obtained from a mastitic milk culture: the microorganisms associated with the infection might be shed intermittently; or the number of viable bacterial cells is small; finally, the cow’s immune system might have eliminated the pathogen, and the observation of abnormal milk could be a consequence of the inflammatory process that occurred during destruction of the pathogen^42^. Nevertheless, mastitis has also been reported to be caused by mechanical or chemical injury, as well as by non-bacterial infectious agents such as viruses^43^ and yeasts^44^. Although infrequent, it is important to acknowledge that a portion of these culture negative mastitis cases can be result of a viral infection playing a role in clinical mastitis. In fact, the historical role of viruses in mastitis might have been underestimated, given that the practice of laboratory diagnosis of viruses in mastitis cases is unusual^43^.

In our study, mastitic quarters yielding a negative aerobic result differed in bacterial load compared to their healthy counterparts. This is a very interesting finding, as we were not able to identify in the microbial profiles any specific bacterial family that could be associated with these mastitis cases. The identification of a higher bacterial load not linked to a specific group of pathogens might indicate that dysbiosis occurs not only with changes in the composition of the mammary microbiota, but also with a simple nonspecific increase of intramammary bacterial load, leading to clinical signs of mastitis. It is true that the number of 16S rRNA copies in the genome is variable, which can impact bacterial community analysis^45^; however, the quantification of 16S rRNA gene has been proved to be useful as a proxy for estimating bacterial load^46^. In our study, mastitic quarters exhibited significantly lower microbial diversity upon diagnosis compared to healthy quarters, which could indicate that fewer microbes were dominating the milk microbiome. Our results are in line with those of Kuehn *et al*.^10^, who identified that the microbiome of mastitic quarters is less diverse than healthy ones in culture-negative mastitis cases. Recent work by Falentin *et al*.^47^ has raised an interesting discussion when it comes to microbial diversity, dysbiosis, and disease. The investigators demonstrated that animals presenting normal milk at sampling, but with different histories in regards of clinical and subclinical mastitis had remarkably different bacterial diversity, as well as an altered microbial profile far from an episode of clinical mastitis. Research is warranted to determine the relationship between changes of the mammary microbiota and timing of clinical mastitis, and elucidate if a shift in the microbial profile predisposes to clinical mastitis, or if an active colonization of a rather normal microbiome is to be held accountable for both the clinical episode and the lasting effect on the alteration of the milk microbiome. Koskinen *et al*.^48^ evaluated the use of a pathogen-specific real-time PCR assay for identification of mastitis bacteria and reported that 76% of culture-negative clinical mastitis samples were positive for various mastitis pathogens, including members of the Streptococci, Staphylococci and Enterobacteriaceae families. However, it has previously been reported that such bacteria are found in the microbiome of healthy milk of both humans and cows^11^,^18^,^49^. Although infrequent, mastitis caused by different species of *Mycobacterium* has been reported in bovines, alpacas and dogs^50^,^51^,^52^,^53^. *Mycobacterium* is often misidentified as a negative culture due to its slow growth characteristics and because it is a facultative anaerobic microbe^50^,^54^. In our results, we observed a non-significant increase in the relative abundance of Mycobacteriaceae in mastitic animals yielding negative aerobic culture results. Identifying which microorganisms are associated with culture-negative mastitis does not justify the use of antimicrobial treatment; however, it does shed light on the bacterial etiology of the disease, facilitating decision-making regarding mastitis prevention strategies.

Differences in the microbiome of healthy and mastitic milk samples have also been reported for cows^9^,^10^,^11^ and humans^18^,^38^. However, a unique feature of the research presented here is that we used a controlled randomized clinical trial approach to longitudinally describe the differences between milk from mastitic mammary glands and from healthy ones and the impact of antibiotic therapy on the microbiome from the onset of disease until its resolution. To our knowledge, this is the first study to longitudinally evaluate the effect of antimicrobial therapy using the combination of quantitative PCR and next-generation DNA sequencing in dairy cows. Bovine milk, similarly to human milk, exhibits a complex and dynamic microbial ecology^9^,^10^,^11^,^18^,^38^,^55^. Nevertheless, significant efforts have been recently undertaken using culture-independent techniques to evaluate the effects of antibiotic therapy in swine^56^, horses^57^, gorillas^58^, and humans^59^,^60^,^61^,^62^,^63^.

Antimicrobial use in the food industry could potentially impact human health, warranting its judicious use^64^,^65^. Ceftiofur is the only FDA-approved third-generation cephalosporin for use in food-producing animals and has been classified by the World Health Organization as one of the critically important antimicrobials for human medicine^66^. In summary, our work corroborates the existing literature and also provides novel evidence that the use of intramammary ceftiofur therapy for the treatment of mild and moderate cases of *E. coli*-caused and culture-negative mastitis is ineffective. More importantly, it suggests that antimicrobial stewardship in food animals can be achieved in certain situations without compromising the health of the animals. Additionally, the combined use of quantitative PCR and sequencing of the 16s rRNA gene is an effective approach to evaluate the usefulness of antibiotic therapy.

## Additional Information

**How to cite this article**: Ganda, E. K. *et al*. Longitudinal metagenomic profiling of bovine milk to assess the impact of intramammary treatment using a third-generation cephalosporin. *Sci. Rep.* **6**, 37565; doi: 10.1038/srep37565 (2016).

**Publisher’s note:** Springer Nature remains neutral with regard to jurisdictional claims in published maps and institutional affiliations.

## Supplementary Material

Supplementary Material

## Figures and Tables

**Table 1:** Effects of intramammary treatment with ceftiofur hydrochloride on clinical mastitis cure in lactating dairy cows.

Parameter	Clinical cure			Bacteriological cure^1^		
	Control	Ceftiofur	*P*	Control	Ceftiofur	*P*
	% (n/n)			% (n/n)		
Cure on day 10 and 14						
Gram negative	73.9 (17/23)	75.0 (18/24)	0.93	82.6 (19/23)	79.2 (19/24)	0.76
*Escherichia coli*	75.0 (15/20)	70.0 (14/20)	0.72	85.0 (17/20)	80.0 (16/20)	0.67
*Klebsiella* spp.	66.7 (2/3)	100.0 (2/2)	0.36	66.7 (2/3)	100.0 (2/2)	0.36
*Pseudomonas* spp^2^	—	100.0 (2/2)	—	—	50.0 (1/2)	—
Negative culture^3^	76.5 (13/17)	81.3 (13/16)	0.73	NA	NA	NA
Overall	75.0 (30/40)	77.5 (31/40)	0.79	82.6 (19/23)	79.2 (19/24)	1.00

^1^Based on standard laboratory culture methods for identification of milk pathogens.

^2^All cows diagnosed with *Pseudomonas* spp. by laboratory culture were assigned to the Ceftiofur group; thus, evaluation of treatment effect was not possible.

^3^Evaluation of bacteriological cure is not applicable to cows with negative culture. NA = non-applicable.
