# Supplementary material for: Longitudinal metagenomic profiling of bovine milk to assess the impact of intramammary treatment using a third-generation cephalosporin
Source: Sci Rep. 2016 Nov 22;6:37565. doi: 10.1038/srep37565 (PMC5118806; doi:10.1038/srep37565)
Supplement: Supplementary Material [file srep37565-s1.pdf]

Longitudinal metagenomic profiling of bovine milk to assess the impact of intramammary treatment using a third-generation cephalosporin

Supplementary Material

Erika K. Ganda[1], Rafael S. Bisinotto[1,2], Svetlana F. Lima[1], Kristina Kronauer[1], Dean H. Decter[1], Georgios Oikonomou[1,3], Ynte H. Schukken[1], and Rodrigo C. Bicalho[1,*]

[1] Department of Population Medicine and Diagnostic Sciences, College of Veterinary Medicine, Cornell University, Ithaca, NY, USA

[2] Current address: Department of Veterinary Population Medicine, College of Veterinary Medicine, University of Minnesota, St. Paul, MN, USA

[3] Epidemiology and Population Health, Institute of Infection and Global Health, University of Liverpool, Liverpool, UK

[*] Correspondence and requests for materials should be addressed to R.C.B. (email: rcb28@cornell.edu).

FIG S1. Comparison of the microbiome from quarters with clinical mastitis associated with *Pseudomonas* spp. and healthy quarters (i.e. reference for calculation of fold change). Size of the circle is proportional to the overall prevalence of each family. Color of the circle is associated with effect size. The graph plots log fold change in 16S rRNA gene abundance in mastitic relative to healthy control quarters versus false discovery rate (FDR) logWorth (i.e. -log10P). P-values are adjusted for FDR. The dashed line represents adjusted *P*-value = 0.05.

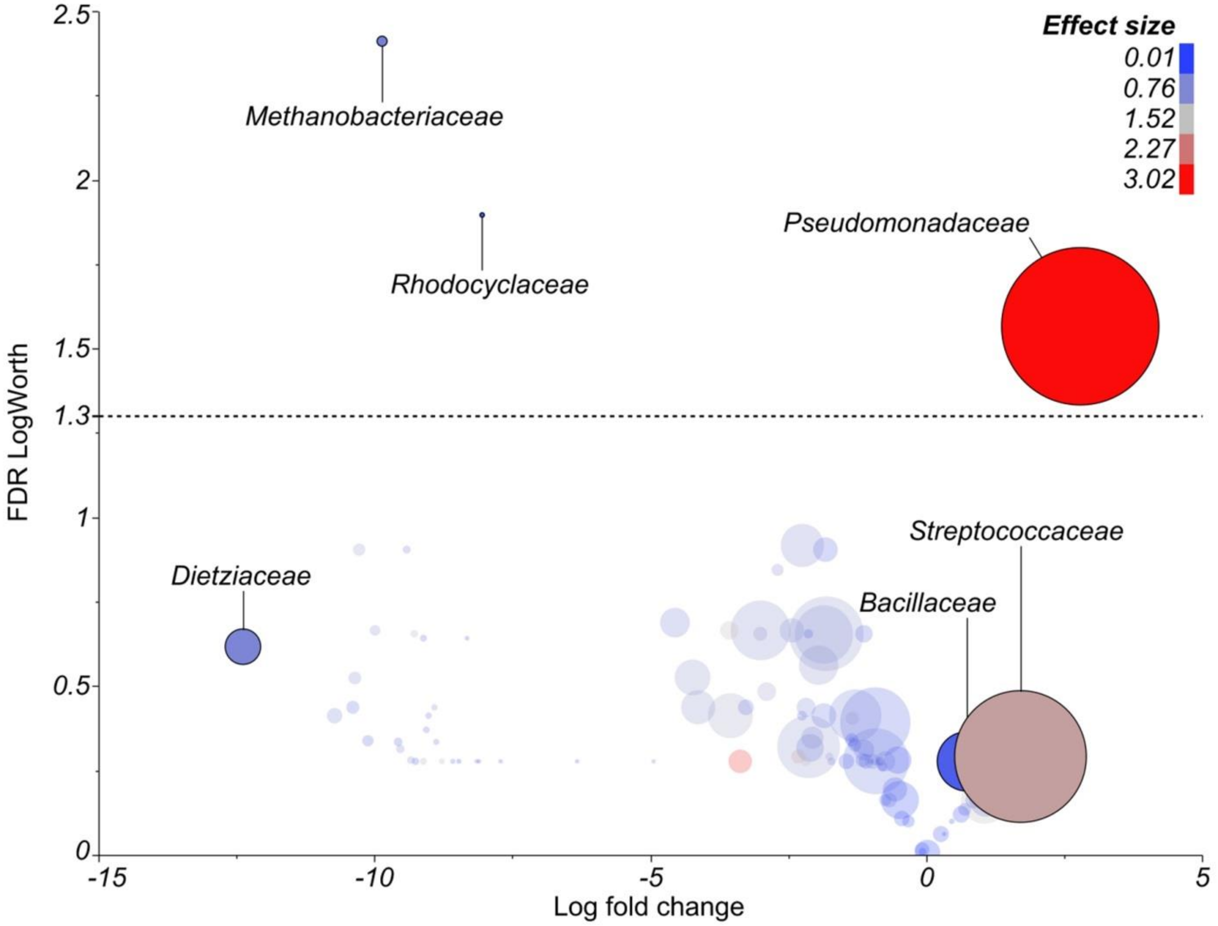

FIG S2. Effect of clinical mastitis and intramammary treatment with ceftiofur hydrochloride on richness of the milk microbiome in cows with clinical mastitis associated with *Escherichia coli* (a) and in cows with clinical mastitis associated with a negative culture (b).

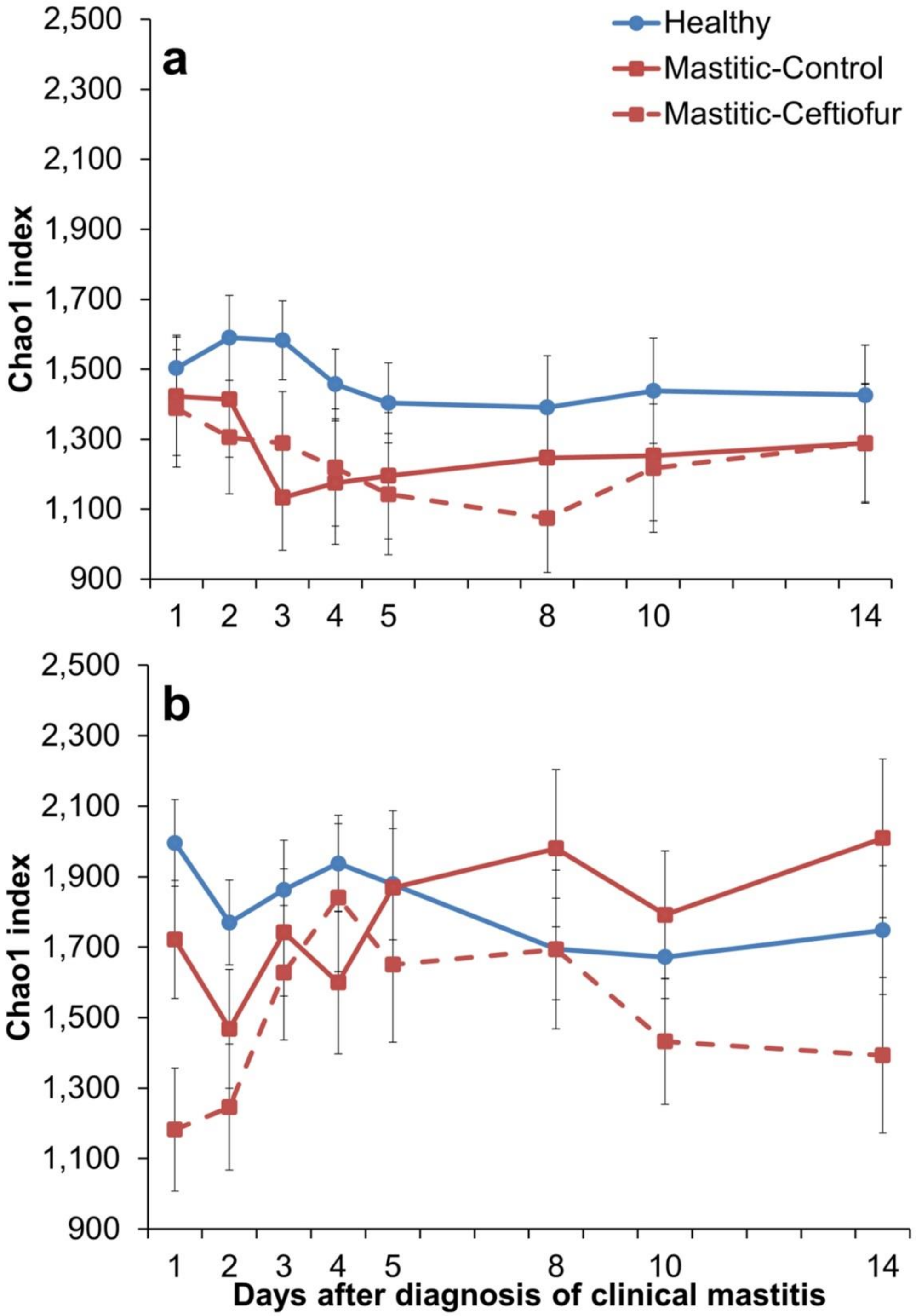

FIG S3. Comparison of the microbiome from quarters with clinical mastitis associated with *Klebsiella* spp. and healthy quarters (i.e. reference for calculation of fold change). Size of the circle is proportional to the overall prevalence of each family. Color of the circle is associated with effect size. The graph plots log fold change in 16S rRNA gene abundance in mastitic relative to healthy control quarters versus false discovery rate (FDR) logWorth (i.e. -log10P). P-values are adjusted for FDR.

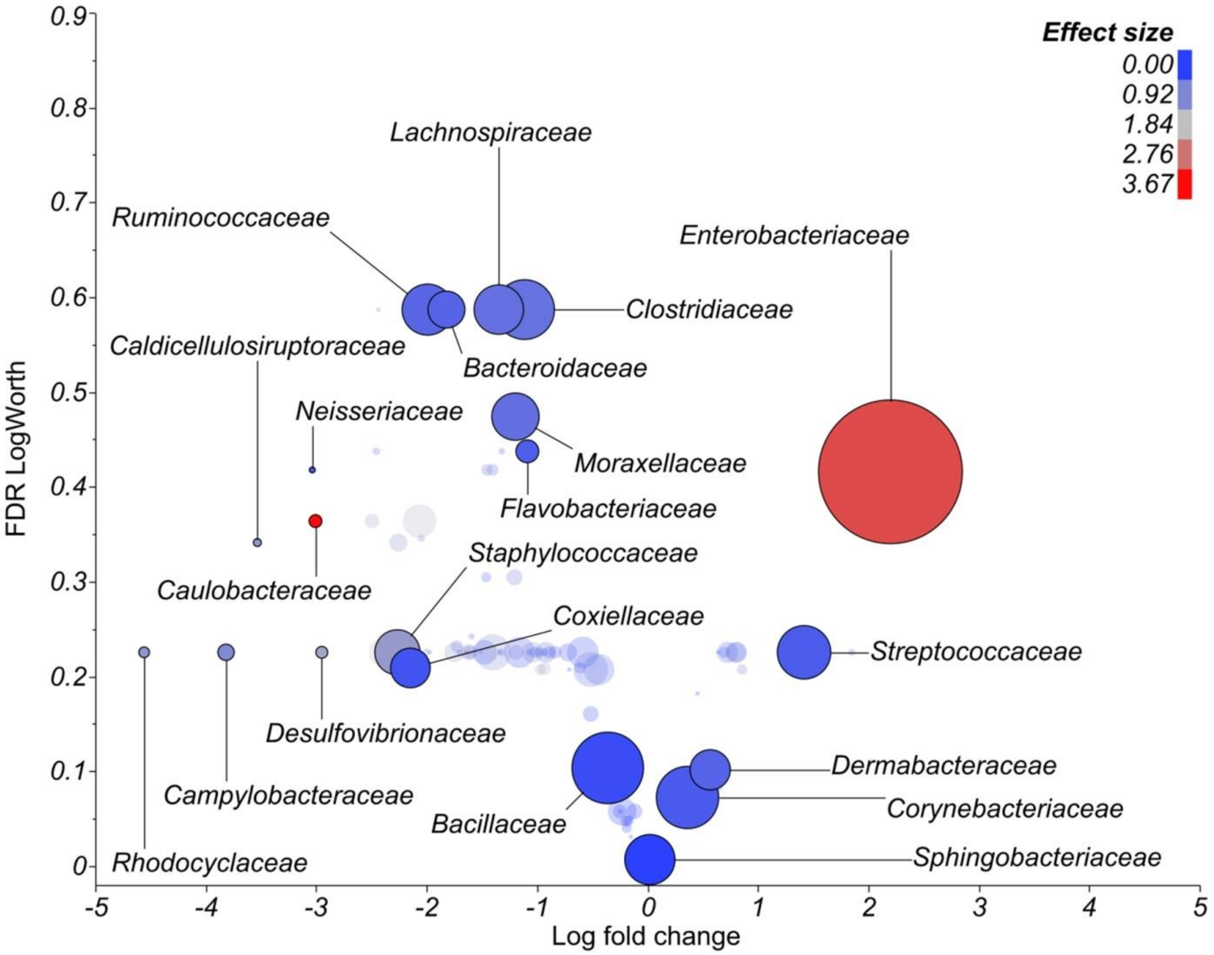

FIG S4. Effect of clinical mastitis and intramammary treatment with ceftiofur hydrochloride on diversity (a) and richness (b) of the milk microbiome in cows with clinical mastitis associated with *Klebsiella* spp.

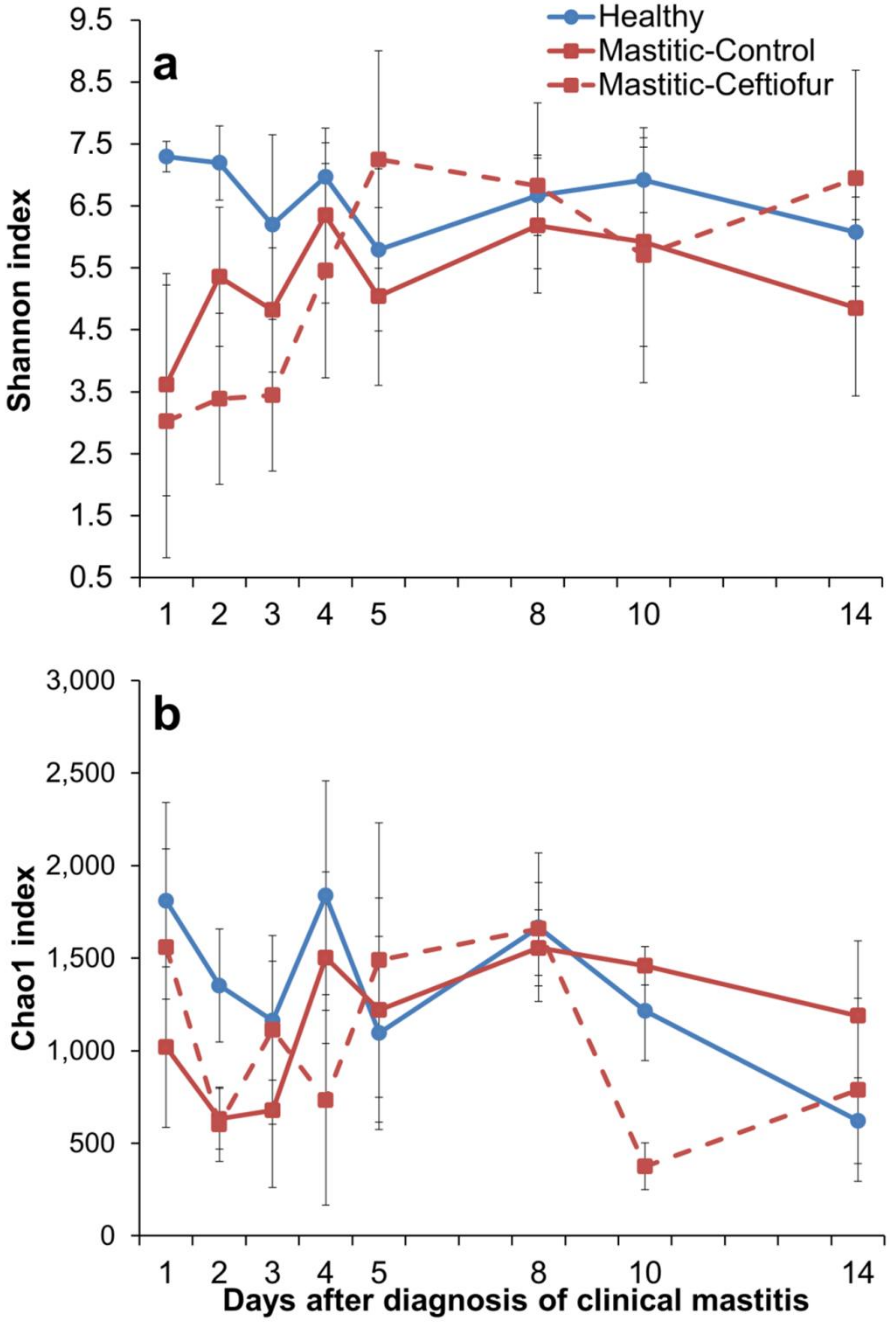

FIG S5. Changes in relative abundance of the 25 most prevalent families in milk from quarters with clinical mastitis associated with *Klebsiella* spp. and healthy quarters. Numbers indicate day after diagnosis of clinical mastitis.

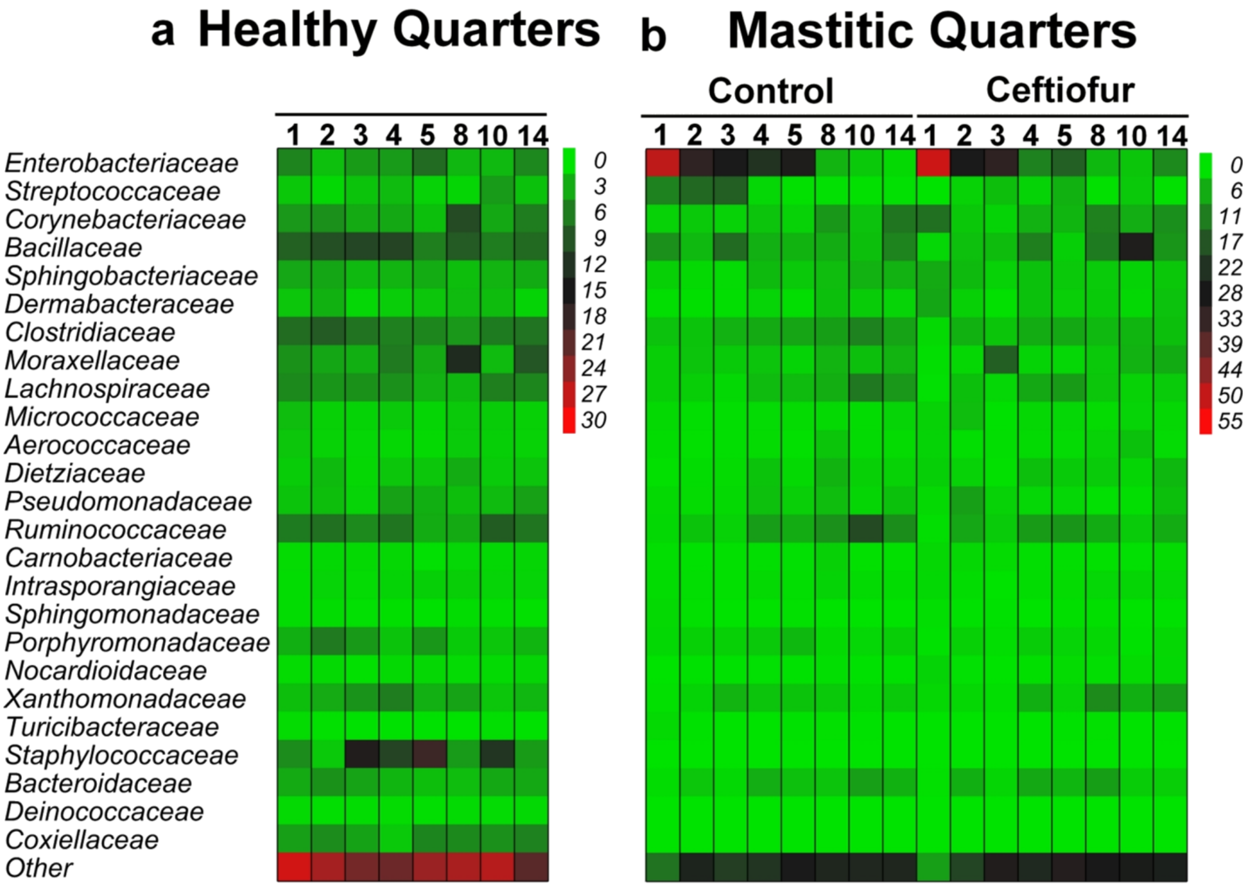

FIG S6. Effect of intramammary treatment with ceftiofur hydrochloride (day 1) on the relative abundance of Enterobacteriaceae in cows with clinical mastitis associated with *Klebsiella* spp. TRT = effect of treatment; Day = effect of time; T×D = interaction between treatment and day.

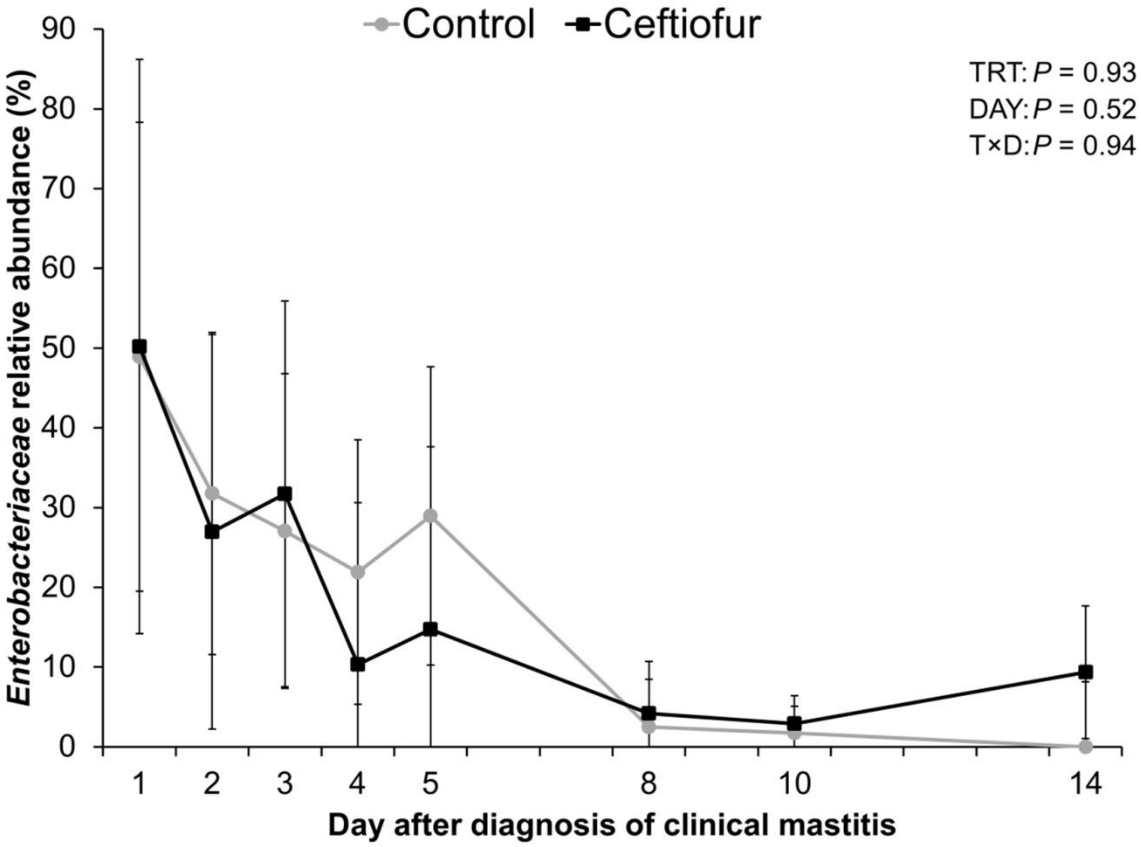

FIG S7. Effect of intramammary treatment with ceftiofur hydrochloride (day 1) or cure on the relative abundance of Enterobacteriaceae and Shannon diversity index in cows with clinical mastitis associated with *Escherichia coli*. (a) Effect of intramammary treatment with ceftiofur hydrochloride (day 1) on the relative abundance of Enterobacteriaceae in cows with clinical mastitis associated with *E. coli*. Effect of eventual bacteriological cure on the relative abundance of Enterobacteriaceae in cows with clinical mastitis associated with *E. coli* on control cows (c) and treated cows (e). Effect of cure on the Shannon index in cows with clinical mastitis associated with *E. coli* (b), on control cows (d) and treated cows (f). Asterisks represent significant differences at $\alpha = 0.05$ between groups within the same study day.

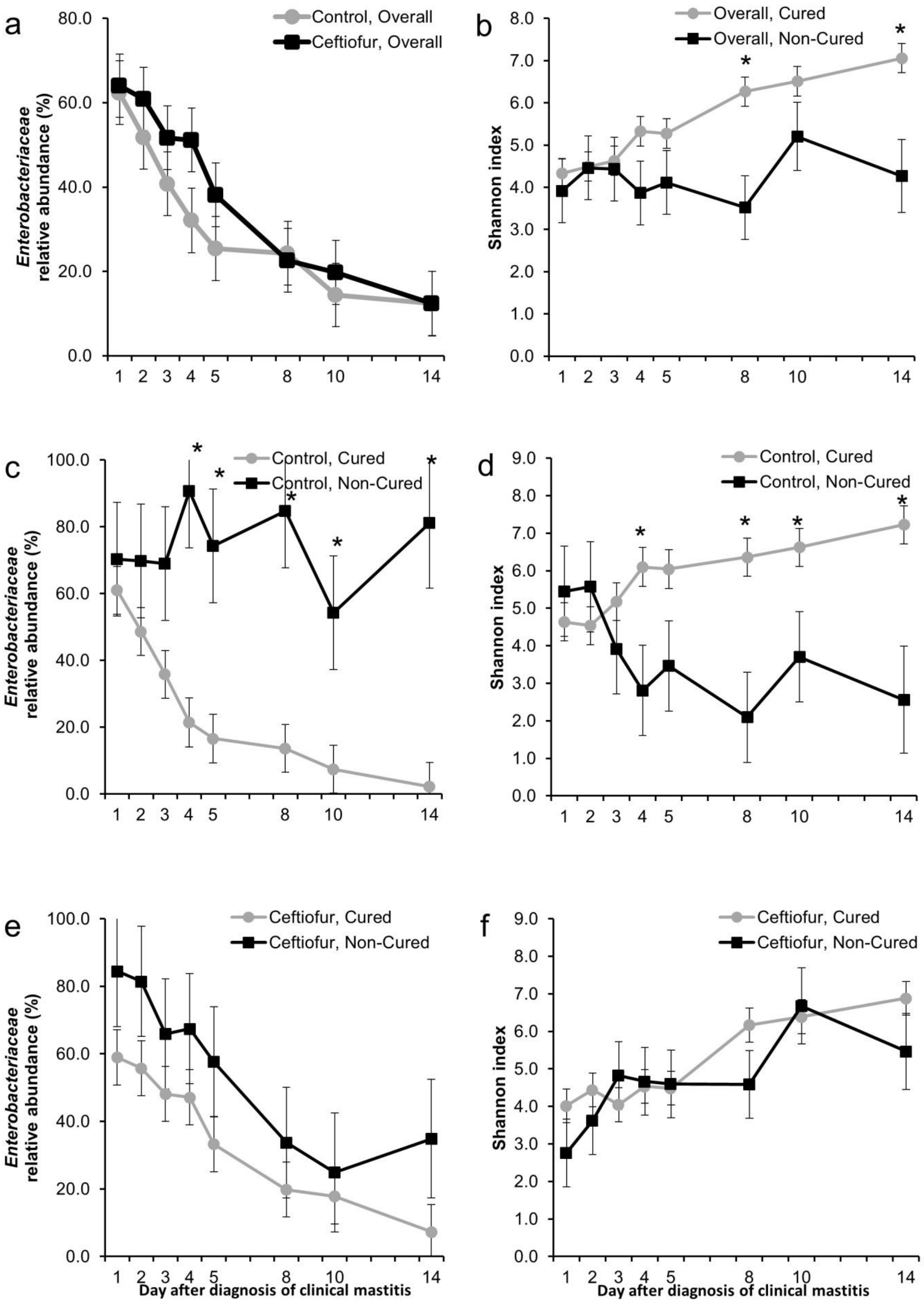

a
Control, Overall
Ceftiofur, Overall
Enterobacteriaceae relative abundance (%)
b
Overall, Cured
Overall, Non-Cured
Shannon index
c
Control, Cured
Control, Non-Cured
Enterobacteriaceae relative abundance (%)
d
Control, Cured
Control, Non-Cured
Shannon index
e
Ceftiofur, Cured
Ceftiofur, Non-Cured
Enterobacteriaceae relative abundance (%)
Day after diagnosis of clinical mastitis
f
Ceftiofur, Cured
Ceftiofur, Non-Cured
Shannon index
Day after diagnosis of clinical mastitis
